# Why Is It so Hard to Do Good Science?

**DOI:** 10.1523/ENEURO.0188-18.2018

**Published:** 2018-09-06

**Authors:** Ray Dingledine

**Affiliations:** 1 Department of Pharmacology, Emory University School of Medicine, Atlanta, GA 30322

**Keywords:** Bayesian, cognitive psychology, decision making, premortem, reproducibility, rigor

## Abstract

“Good science” means answering important questions convincingly, a challenging endeavor under the best of circumstances. Our inability to replicate many biomedical studies has been the subject of numerous commentaries both in the scientific and lay press. In response, statistics has re-emerged as a necessary tool to improve the objectivity of study conclusions. However, psychological aspects of decision making introduce preconceived preferences into scientific judgment that cannot be eliminated by any statistical method. The psychology of decision making, expounded by Kahneman, Tversky, and Thaler, is well known in the field of economics, but the underlying concepts of cognitive psychology are also relevant to scientific judgments. I repeated experiments carried out on undergraduates by Kahneman and colleagues four to five decades ago, but with scientists, and obtained essentially the same results. The experiments were in the form of written reactions to scenarios, and participants were scientists at all career stages. The findings reinforce the roles that two inherent intuitions play in scientific decision making: our drive to create a coherent narrative from new data regardless of its quality or relevance and our inclination to seek patterns in data whether they exist or not. Moreover, we do not always consider how likely a result is regardless of its *p* value. Low statistical power and inattention to principles underpinning Bayesian statistics reduce experimental rigor, but mitigating skills can be learned. Overcoming our natural human tendency to make quick decisions and jump to conclusions is a deeper obstacle to doing good science; this too can be learned.

## Significance Statement

Societal approaches to improving the rigor and reproducibility of preclinical biomedical science have largely been technical in nature with a renewed focus on the role of statistics in good experimental designs. By contrast, the importance of preconceived notions introduced by our very human nature has been underappreciated for their influence on scientific judgments. Explicitly recognizing and addressing these cognitive biases, and including such strategies as carrying out a “premortem” before embarking on new experimental directions, should improve scientific judgments and thereby improve the quality of published findings, eventually boosting public confidence in science.

## Introduction

Most failures in advanced (phase 3) clinical trials for small molecule drug candidates can be traced to insufficient efficacy (Hay et al., 2014). Although clinical trial design fettered by corporate needs and the meager predictive value of many preclinical animal models undoubtedly contribute to these failures, especially for some neuropsychiatric disorders, there is a growing realization that suboptimum experimental practices in preclinical research are also a prominent cause of the problem. Over-confidence in favorable *p* values, low statistical power, and the waning of traditional good experimental design practices such as blinding and inclusion of both positive and negative controls have all been highlighted as likely sources of misleading preclinical research (Ioannidis, 2005, 2018; Begley and Ellis, 2012; Button et al., 2013; Marino, 2014; Halsey et al., 2015; Steward, 2016; Colquhoun, 2017). Recent articles in the lay press make the general public aware of this issue (Leaf, 2013; The Economist, 2013, 2018; Carroll, 2017). Poor reproducibility of scientific research undermines public confidence in science and leads to waste of resources when investigators attempt to replicate and extend fallacious findings.

The scientific community is responding vigorously to this growing “crisis of confidence” in the reliability of preclinical science. Universities are reemphasizing a working knowledge of statistics that extends beyond “plug and play” software. Professional societies such as the Society for Neuroscience are providing web-based resources for enhancing the rigor and transparency of research (https://www.sfn.org/news-and-calendar/news-and-calendar/news/professional-development/virtual-conference-scientific-rigor-transparency-science). Journals are providing checklists meant to enhance transparency and, in some cases, employing statisticians on their editorial boards to check the veracity of findings. A checklist has been introduced to help recognize research that might have one or more procedural flaws (Begley, 2013). Finally, the National Institutes of Health has introduced mandatory sections in grant proposals that are meant to improve rigor (https://grants.nih.gov/reproducibility/index.htm). This is all good and helpful, but renewed attention to statistics and good experimental design practices will not, by themselves, solve this problem. Inherent in the goal of improving the reproducibility of preclinical science is the need to change behavior. Accepting the value of statistics or proper experimental design is rarely enough to bring about the desired change. In this regard, science can benefit from insights gleaned in the fields of investing, finances and marketing from considerations of how people arrive at judgments and make decisions.

In the late 1960s, two young psychologists, Daniel Kahneman and Amos Tversky, initiated a series of experiments designed to understand how people make decisions when presented with fragmentary information of uncertain relevance. Thus seeded the field that evolved into behavioral economics (Thaler, 2016). Their experiments took the form of questions that subjects would answer, and a comparison of their answers with statistically-apt outcomes. Among their major findings was that preconceived notions and unconscious emotions often dominate decision making when one is presented with new or unfamiliar data (Tversky and Kahneman, 1974; Kahneman and Tversky, 1979). In their explanation, the nature of decision making often involves over-reliance on intuition, to a degree not explicitly recognized, and underappreciation of the role of chance in events. Kahneman (2011) describes two approaches to decision making, unconscious, intuitive and reflexive, or conscious, deliberate and reflective, which he terms fast and slow thinking, respectively. Fast thinking is easily influenced by cognitive biases, whereas slow thinking is more resistant. A major outcome of their work is the realization that emotion-driven preconceptions and hopes play a large role in financial, investing, political, and economic decisions, for example, the notions that we are all good investors (Belsky, 2016), that trade wars are quickly winnable (North, 2017), or in the present context, that our scientific projects will proceed without insurmountable hitches.

Findings from cognitive psychology have been applied to fields as disparate as hostage negotiation (Voss, 2016) and healthcare system planning (Harrison et al., 2018). Science prides itself on objective thinking; I wondered whether insights gleaned from cognitive psychology were relevant to decisions made by scientists. Kahneman and Tversky’s subjects were mostly undergraduate students. My notion, and the hypothesis I tested, was that a scientifically literate population would respond more objectively to the survey questions originally posed in the 1960s and 1970s. I was wrong.

A three-page questionnaire consisting of five questions was designed based on the projects originally conducted by Kahneman and Tversky as described below. The surveys were distributed to research personnel in three basic science departments, consisting of faculty, postdoctoral fellows, graduate students, and senior research technicians. Adult English‐speaking scientists were the intended target population without regard for gender or ethnicity. Voluntary participants then completed the survey anonymously, estimated to require <15 min, and the surveys were returned to the author for analysis. There were 44 respondents out of ∼70 surveys distributed. The protocol was approved by Emory’s IRB.

## Prologue

I began this project by seeking the opinions of colleagues about difficulties they encounter in their day-to-day pursuit of biomedical research. When asked what, aside from the scientific project itself, challenges them the most in their research, a sampling of postdoctoral fellows, students, and research technicians had several reactions:

- The PI can become distant from the project; expectations and reality diverge.
- Lab members without sufficient training can be expected to work on the project.
- Reproducing a method developed in another lab can be difficult.
- Getting all parameters of an experiment under control can be problematic.
- Perverse publishing incentives are rampant – quantity and impact factor over quality.
- Appropriate controls can sometimes be difficult to identify.
- Rigorous experiments can be labor intensive and expensive.

Regarding the last point, it is helpful to separate experiments into two categories (cf. Kimmelman et al., 2014). Exploratory experiments are quick and sometimes without all necessary controls, are often underpowered, done to explore and adjust experimental parameters to sharpen the hypothesis, and do not usually incorporate a blinding step in analysis. Low statistical power and the absence of a blinding step result in many false positives and findings that inevitably regress toward the mean during in-house replication attempts. The large majority of preclinical research falls into this category, and much of biomedical graduate training emphasizes this approach. On the positive side, experimental results from disparate approaches that test the same hypothesis, each of which is underpowered statistically, can be combined to produce a coherent conclusion. In the absence of a blinding step, however, unconscious bias still reduces confidence. Definitive experiments, by contrast, require the experimenter and/or data analyzer to be unaware of treatment groups, involve a larger sample size and carefully restrict the number of comparisons to be made. Often the results and conclusions will form the basis of a larger effort (e.g., a multi-year grant application, a Ph.D. thesis project, or a clinical trial). Definitive experiments can be expensive, both in time and money, but the results have a better chance of standing the test of time. Most of what follows is applicable to both categories of experiment.

The informal responses in the bulleted list above indicate scientists are well aware of logistic impediments to doing good science. No one, however, raised the possibility that cognitive biases might present difficulties. I repeated several experiments that Kahneman and Tversky conducted on undergraduates in the 1960s and 1970s to determine if objectivity is enhanced in a scientifically literate population. A set of five survey questions that had been originally designed by Kahneman and Tversky was distributed to colleagues in several basic science departments at Emory University School of Medicine, and the results tabulated. Responses to one of the five questions suggested that its wording was ambiguous, so what follows describes responses to four survey questions. The questions are presented verbatim below.

## The Law of Small Numbers

One survey question asked respondents to consider the following:

“A certain town is served by two hospitals. In the larger hospital about 45 babies are born each day, and in the smaller hospital about 15 babies are born each day. As you know, about 50 percent of all babies are boys, although the exact percentage varies from day to day. Sometimes it may be higher than 50 percent, sometimes lower. For a period of 1 year, each hospital recorded the days on which more than 60 percent of the babies born were boys. Which hospital do you think recorded more such days?”

Forty-four respondents answered as follows:

The larger hospital: 3

The smaller hospital: 18

The hospitals are within 5% of each other: 23.

Approximately 59% of scientists thought the large and small hospitals would be similar or the larger hospital would have more such days. Sampling theory, however, indicates that the smaller hospital would have about twice as many days in which >60% of infants were boys (98 ± 4 vs 48 ± 2 d with *n* = 10 repetitions of a bootstrapped model). Interestingly, 41% of today’s scientists chose the correct answer compared to only 20% of the original sample of 97 undergraduates with little or no background in probability or statistics. The *p* value of 0.041 comparing the two cohorts (Fisher’s exact test) is encouraging of progress until one calculates the statistical power of the comparison, which is only 52% (G*power 3.1.9.2). Low statistical power increases the likelihood of a false positive (Steward, 2016).

In a second question, a group of faculty, postdocs, and graduate students reacted to the following scenario:

“The distribution of newly-diagnosed kidney cancer in the 3,141 counties in the U.S. reveals that the counties with lowest incidence are mostly rural, sparsely populated, and located in traditional Republican states in the Midwest, the South and the West. What do you make of this?”

Non-political answers were (with number of respondents in parentheses if >1):

Better diet (5; eating more greens, less processed foods, organic); poor diagnosis in rural areas (3); lower anxiety and stress (3); rare forms of cancer less likely if fewer people (2); healthier lifestyle; less medical care; early mortality due to other causes; counties are undersampled; smaller population leads to lower numbers with cancer; smaller sample size means greater variance.

A separate group was presented with the same scenario, except the word “lowest” was replaced by “highest.” Their responses:

Low access to health care or preventive care (7); pesticides or other contaminants in groundwater are carcinogenic (6); increased recent screening (3); low access to healthy foods (2); socioeconomic status leads to higher stress; education level; ethnicity; less healthy lifestyle.

**Figure 1.**
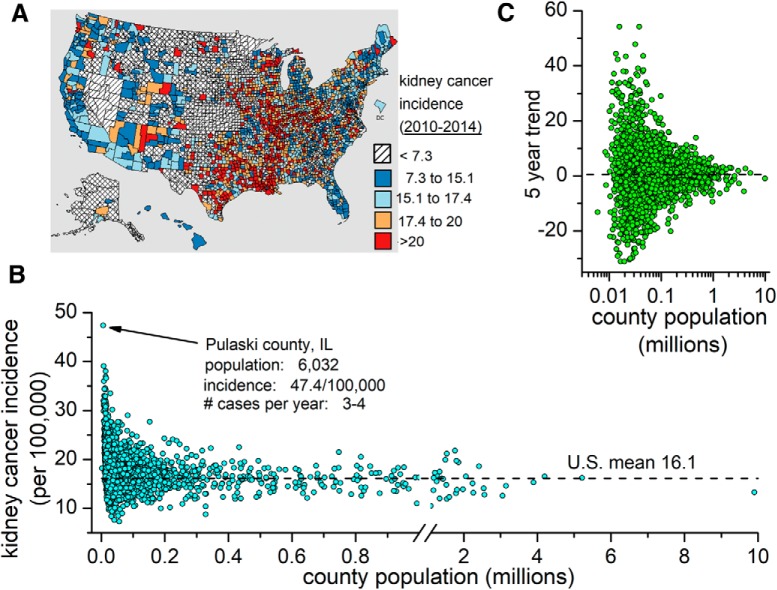
Low sample size produces high variance. ***A***, Map of the average kidney cancer incidence in each of the 3141 counties of the United States between 2010 and 2014. ***B***, Plot of incidence as a function of county population. ***C***, Five-year trend in incidence versus population. Data from the CDC National Program of Cancer Registries.

It is interesting that stress, diet, and lifestyle feature prominently in both scenarios but are used to reach opposite conclusions. Both scenario statements are correct; moreover, a county that has a high incidence one year can have low incidence the next. The two words in both scenarios most relevant to understanding the nature of the problem are “sparsely populated,” as shown in Figure 1; low *n* leads to high variability.

Both of these examples demonstrate an inherent belief in the Law of Small Numbers (Tversky and Kahneman, 1971), which is the tendency to accept data from a small sample as representative of the whole population. This inclination likely contributes to the willingness to do underpowered experiments and accept the outcome if one is lucky enough to have a favorable *p* value. These data are congruent with the proposal (Kahneman, 2011) that our tendency to build a narrative around new data, however irrelevant, is strong and can prevent us from getting at the underlying drivers of our observations.

## Intuitive Pattern Seeking

People seek patterns, as illustrated by the following examples. A group of scientists were asked to consider three possible sequences of the gender of eight infants born in a row at the same hospital:

BBBBGGGG

GGGGGGGG

BGBBGBGB

They were asked: “Do these sequences seem equally likely?”

Yes: 20 respondants

No: 24 respondants

**Figure 2.**
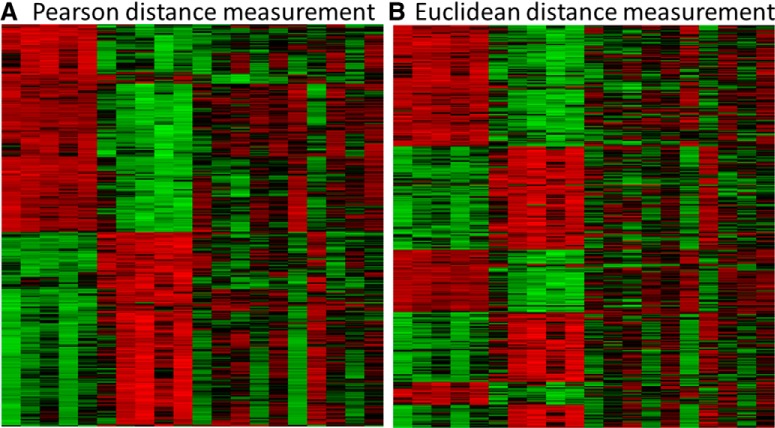
Microarray clustering procedure drastically alters the visual pattern. The data were from dentate granule cells provided by seven laboratories and consist of median log2 expression values of 398 genes that were differentially expressed (FDR < 0.05) with ≥2-fold expression change between control rats and rats in three status epilepticus (SE) models at different times after SE (Dingledine et al., 2017). Complete linkage and row (gene)-clustering only; the relative positions of columns (treatment groups) in ***A***, ***B*** are unvarying. The only difference in the two procedures was the distance measurement used. The Heatmapper tool was used for clustering and visualization (http://www1.heatmapper.ca/expression/).

Any specific sequence is as likely as any other, but 55% of respondents thought otherwise. Their written comments indicated that many thought eight girls in a row was extremely unlikely, which is true (probability = 2E-8 or 0.4%) but no less likely than the specific sequence below it. We all try to look for patterns in our data, whether they exist or not. Random events can produce runs of seeming regularity (Taleb, 2004), and our pattern-seeking human nature can easily reach a false conclusion. It can be particularly difficult to trust the eye when evaluating large datasets, because seemingly small differences in analysis procedure can result in substantial visual differences in patterns, as illustrated by different clustering algorithms of the same microarray dataset in Figure 2. In Figure 2*A*, the data appear to segregate into two main groups, whereas in Figure 2*B*, the same data collect into six groups.

**Figure 3.**
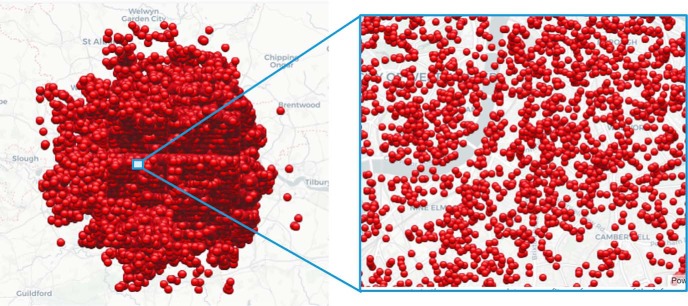
Pockets of seeming regularity in a random process. The left panel shows the location of each of the bombs dropped on London during the eight-month blitzkrieg of 1940–1941. Although the city was blanket-bombed, there were isolated neighborhood-size areas in which no bombs fell as shown on the right panel (from http://www.dailymail.co.uk/sciencetech/article-2243951/The-astonishing-interactive-map-EVERY-bomb-dropped-London-Blitz.html).

A final, somewhat treacherous, example of pattern seeking is illustrated by the spatial pattern of bombs dropped on London during the eight-month-long blitz in 1940–1941. Although when viewed from a distance the bombing pattern appears very dense (Fig. 3, left), a detailed view of smaller regions reveals scattered neighborhoods that were spared throughout the eight-month period (Fig. 3, right). Reports at the time speculated that the bombing pattern was not random (Feller, 1950, p 120) and that German spies might have resided in the spared neighborhoods (Kahneman, 2011, p 116), a conclusion that could only have been socially disruptive during a trying time. However, a binomial analysis of the spatial pattern of impact sites was fully consistent with a random pattern of bomb locations (*p* = 0.88 by χ^2^; Clarke, 1946). Taken together, these examples support a tendency of scientists and non-scientists alike to find patterns in data.

## Ignoring the Likelihood of a New Result

The base rate is the probability that a member of a specific population will have a certain characteristic, assuming that we know nothing else about this individual. Does the base rate influence decisions when new data are introduced? A group of 43 scientists considered the following scenario:

Chris is of high intelligence, although lacking in true creativity. He has a need for order and clarity, and for neat and tidy systems in which every detail finds its appropriate place. His writing is rather dull and mechanical, occasionally enlivened by somewhat corny puns and flashes of imagination of the sci-fi type. He has a strong drive for competence. He seems to have little feel and little sympathy for other people, and does not enjoy interacting with others. Self-centered, he nonetheless has a deep moral sense.

Respondents were asked to rank order nine fields by the likelihood that Chris is in that field (1 = most likely, 9 = least likely). Forty-three scientists replied (Table 1).

**Table 1.** Failure to take employment numbers into account when guessing professional field.

2018Rank	Mean score	Field	2016 United States employment*
1	2.7	Computer science	4,165,140
2	3.0	Engineering	2,499,050
3	3.5	Physics or biology	150,970
4	4.4	Library science	222,760
5	4.8	Law	1,075,520
6	5.5	Business administration	14,371,980
7	6.3	Healthcare	12,361,980
8	7.0	Humanities and education	12,928,630
9	7.9	Social sciences and social work	2,264,070

*Bureau of Labor Statistics.

**Figure 4.**
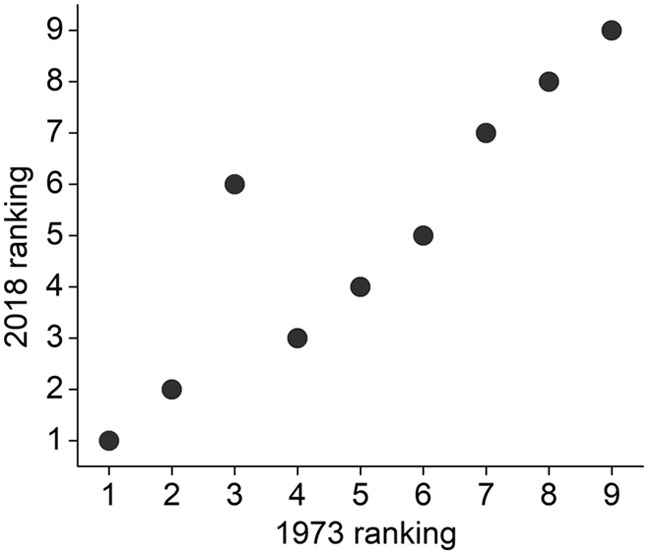
Similarity of judgments in populations of undergraduates and scientists separated by 45 years.

There is no right or wrong answer to this puzzle of course, but even a cursory perusal of the results suggests that the respondents paid little attention to, or were unaware of, the base rates of employment; the four fields deemed least likely have, together, approximately six-fold higher employment than the four deemed most likely. A full 67% of respondents felt that Chris was more likely to be in library science than in business administration, a field that has 64-fold higher employment. Interestingly, the rank order of fields was nearly identical to that found when the study was originally done, although the responding cohorts were as different as undergraduates and scientists, and the tests were separated by 45 years (Fig. 4; Kahneman and Tversky, 1973).

**Figure 5.**
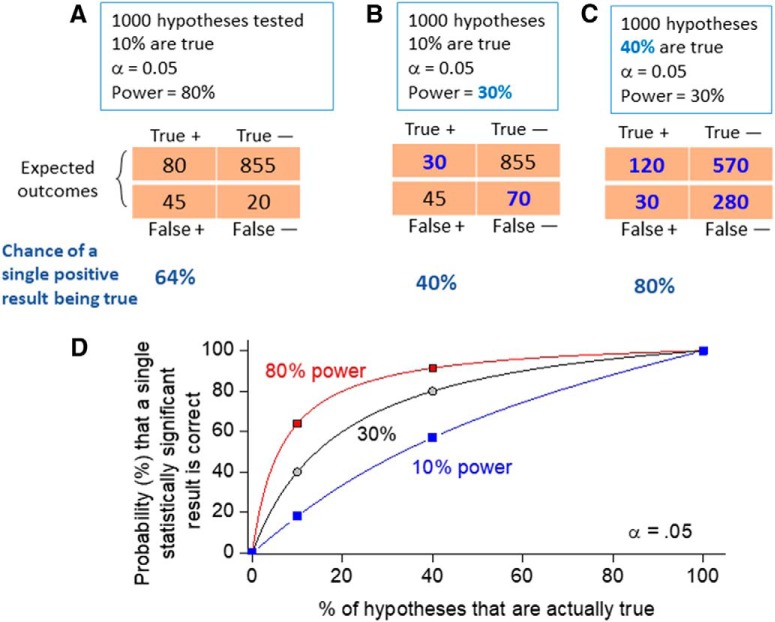
Demonstration of the value of a Bayesian approach when interpreting a single experiment. ***A***, Consider the scientist facing the challenge of testing 1000 hypotheses, for example, in a proteomics or drug screening experiment, in which each protein measured or compound assayed tests the hypothesis that the protein expression is changed or the compound is active. Without knowledge of the target class only a small fraction of these hypotheses (say 10%) are true. If the power of the experiment is 80%, 80 hypotheses will be correctly identified as true (marked “true +”) and 20 will be false negatives (“false –”) as shown in the expected outcomes table. Of the 900 remaining false hypotheses, 45 will be incorrectly ascribed as being true (“false +” in the table) with α = 0.05, and 855 will be correctly ascertained as negatives (“true –”). Thus, the chance of correctly interpreting a positive outcome (equivalent to precision in a ROC analysis) is only 64% (=80/125). ***B***, If the statistical power of the experiment is 30%, the chance of correctly interpreting a positive result drops to only 40% (=45/75). Blue in the outcomes table represent numbers that have changed from the previous panel. ***C***, Now imagine that, with experience, one is working in a more restricted target space in which 40% of hypotheses are expected to be true: the chance of correctly interpreting a positive result jumps to 80% even when power = 30% (=120/150). ***D***, A plot of these variables (plus the 10% power case) shows that the chance of correctly interpreting a single outcome depends on α, power, and the a priori probability that the hypothesis is true.

A related problem arises when one does a properly designed experiment and obtains a result with a statistically significant *p* value. The minimalist conclusion is that the null hypothesis is unlikely to be true, although what we are actually interested in is whether our hypothesis is correct. These are two very different statements. Falsifying the null hypothesis does not imply that our hypothesis is correct; other explanations might serve better. What determines whether a statistically significant result is actually correct rather than being a false positive? Traditional Fisherian statistics highlights the role of statistical power and α (the probability of observing that result or more extreme results, assuming the null hypothesis is correct) in estimating the probability that a statistically significant finding is actually correct. However, the thought experiment presented in Figure 5 indicates that α seriously underestimates the proportion of false positive findings which, in the three examples shown, ranges from 20 to 60% as explained below.

We begin by considering an experiment that tests 1000 hypotheses, 100 of which are expected to be true based on our experience. If our experimental design yields 80% power, only 80 of these true hypotheses will be identified as such and the remaining 20 will be missed, i.e., false negatives. With α = 0.05, the remaining 900 hypotheses are also separated into two groups, with 45 (=0.05*900) being false positives and the remaining 855 appropriately identified as “no effect” or true negatives. These predictions are distributed into the outcomes table in Figure 5*A*. Given this situation, the probability that any single hypothesis that tests positive is actually correct is 80/(80 + 45) = 64%, a far cry from the 95% that is often mistakenly assumed with α = 0.05. Thus, the probability of a false positive is 36% in this case, not 5%. Going on, if our experimental design only allowed a statistical power of 30%, as is common in many published neuroscience papers (Button et al., 2013), the predicted outcomes table indicates that the chance of a single measured positive result actually being true is reduced to only 40% (Fig. 5*B*), i.e., a measured positive will be a false positive 60% of the time. Now the critical part. Assume that with increased knowledge of our experimental system we test hypotheses in a restricted space such that now 40% of the hypotheses are expected to be true (Fig. 5*C*). Under these conditions, our chance of correctly identifying a single hypothesis that measured true rises to 80%, i.e., the false positive rate is reduced to 20%, although α and statistical power are unchanged.

Thus, the *p* value, by itself, does not provide good evidence favoring any non-null hypothesis. Your chance of correctly interpreting a statistically significant result depends on α, statistical power, and *the probability of the result based on prior knowledge* (Fig. 5*D*). Similar arguments have been made by Colquhoun (2014) and Button (2016), among others. The prior knowledge component built into a Bayesian framework can build formal confidence in an hypothesis as results from successive experiments are added (Goodman, 1999; van de Schoot et al., 2014; Buchinsky and Chadha, 2017), always recognizing, however, that an hypothesis, no matter how many supportive repetitions exist, can be refuted by a single well-conceived negative finding (Popper, 1959). The major problem with a Bayesian approach is that it is often difficult to estimate the prior probability of a result before the experiment is conducted, although workarounds have been suggested (Matthews, 2001; Held, 2013; Colquhoun, 2017). A proposed alternative to a Bayesian approach would be to accept a result as statistically significant only if *p* < 0.005 rather than *p* < 0.05 (Benjamin et al.,2018; Ioannidis, 2018); this would reduce the false positive rates in Figure 5 to 5.3%, 13%, and 2.4% in panels *A*, *B*, and *C*, respectively, which are more palatable than the typical situations with *p* < 0.05.

## Conclusions and Recommendations

Why is it so hard to do good science? The work of Kahneman, Tversky, and Thaler, supported by the recent tests described here, points out that a major part of the problem is rooted in our human nature, which makes us prone to jump to conclusions. We have tendencies to favor the law of small numbers, to spin narratives out of datasets of questionable relevance, and to seek patterns in noisy data. Kahneman and Tversky’s biases are prominent in the best of us: it is easier, more social, and more comfortable to think intuitively and quickly, whereas rational, contemplative thinking is difficult, can be tiring, and is frequently a solitary exercise. Second, we often have too much confidence in a favorable *p* value, and sometimes do not pay enough attention to whether a new result is likely to be true regardless of statistical significance. One pernicious outcome of this situation is the proclivity for some journals to publish “very unexpected” findings that nonetheless have statistical significance.

The experiments and scenarios described here are relevant to how people evaluate tests of hypotheses, not how we form hypotheses. The critical thinking skills required to rigorously test hypotheses are quite distinct from the cognitive processes involved in the initial generation of hypotheses. It is striking that answers to the survey questions were so similar regardless of whether respondents were undergraduates in the 1960s and 1970s, or scientific investigators engaged this year. Admittedly, the outcome of the experiments reported here only show that scientists exhibit human features and do not directly demonstrate that we are subject to cognitive biases when planning and evaluating scientific projects. This could be the subject of future work that addresses the circumstances under which scientific decisions are influenced, although senior scientists I have spoken with recognize the relevance to their own research.

Deciding when it is allowable to exclude an outlier, how to incorporate both positive and negative controls, how to determine the number of subjects that will provide an appropriate level of confidence in one’s findings, and selection of the proper statistical test, are all important technical skills that can be learned. Circumvention of cognitive biases, however, is also important in most experiments but particularly preclinical animal trials, cell culture experiments, any experiment in which outcomes are compared among two or more experimental groups.

Notwithstanding the above, plenty of excellent science that stands the test of time is reported by reputable journals every month. How can the substantive investigator improve the quality of scientific endeavors, develop more confidence in our findings, and guard against a premature exit from a research career? First and probably foremost, it is helpful to recognize explicitly the human nature features that can lead us astray; we can then attempt to use Kahneman’s “slow thinking” strategy, recognizing and trying to avoid common biases that unhelpfully support our preconceived notions. Second, we could more often employ good experimental practice that avoids the red flags identified by Begley (2013). It can be helpful in this regard to openly separate experiments into exploratory and definitive categories, and to commit to performing a definitive experiment once the parameters are sufficiently well understood. Of course, there is a need to balance this suggestion against resource availability and time deadlines. Incorporating a Bayesian framework into experimental design could also allow quantification of confidence in the outcome of several small experiments that are considered together. With a Fisherian outlook, accepting a result as “statistically significant” only if *p* < 0.005 will also reduce false positives.

Finally, before embarking on a new line of experiments it can be helpful to perform a “premortem,” i.e., assume the experiment failed and list all potential reasons (cf. Klein, 2007). When setting out in an unfamiliar direction, it is too easy to overestimate the benefits and underestimate the chance of failure with its associated costs. A premortem can present the opportunity to improve rather than autopsy a project. During a premortem, a group of participating scientists would likely identify a wider range of potential problems than just one or two scientists. Framing the discussion by assuming the experiment failed can provide a means for the thoughtful yet somewhat timid members of a group to voice their concerns. I have found that this exercise often encourages the addition of new controls that help us understand why the initial experimental outcome was not as hoped, which in turn accelerates progress toward eventual success.

All of these suggestions would have us acting more like the ancient Greek Titan, Prometheus, and less like his brother, Epimetheus. Prometheus has come to symbolize “forethought” and effective planning, with Epimetheus representing “afterthought.” Recall that in the Greek legends, Prometheus helped advance mankind in many ways, whereas Epimetheus, in a rush, married Pandora and helped open her box.

## Synthesis

Reviewing Editor: Margaret McCarthy, Univ of Maryland School of Medicine Decisions are customarily a result of the Reviewing Editor and the peer reviewers coming together and discussing their recommendations until a consensus is reached. When revisions are invited, a fact-based synthesis statement explaining their decision and outlining what is needed to prepare a revision will be listed below. The following reviewer(s) agreed to reveal their identity: Thomas Abrams.

This is an interesting essay that explores why biomedical findings often do not replicate or stand the test of time, as we are increasingly aware. The manuscript raises interesting and provocative questions about how natural tendencies in human cognition may impede objective analysis and decision making in the course of conducting good science. The paper was inspired by the work of Kahneman and Tversky in behavioral economics, who showed that pre-conceived notions, intuitions, and emotions unconsciously influence decisions when people are faced with fragmentary data of unknown relevance. The author provides several examples of how scientists, like the economists in Kahneman's original study, are influenced by an inherent human drive to find patterns when analyzing and interpreting data, and fall prey to a tendency to generalize findings that are based on small sample sizes. Overall, this essay is clearly developed and well written. It should be of substantial interest to a very broad group of neuroscientists, pharmacologists and other biomedical researchers, and particularly useful for graduate students and postdoctoral trainees. The examples presented are very helpful. The novel strategy of carrying out a “premortem” analysis suggested by the author(s) could be extremely beneficial. Below are both general and specific comments for improvement.

1) The major topic of discussion was whether this is a research report, a commentary or an opinion piece. It was generally agreed that this is in the nature of a commentary but that it includes a small amount of data. So while it does offer useful commentary to the community, it is billed and written as though empirical research were conducted (e.g., as a replication of a previous study). It was agreed that the author should revise the way it's written and formatted so that there's truth in advertising--this is a commentary and should therefore not include a Methods and Results section, for example. That said, the reviewers would still like to see a clearer description of the subject population. In some cases it sounds as if faculty participated, but not in other cases. Maybe there could be a small table with numbers. Even if this is a commentary, when data are presented, it's helpful to understand the population of respondents.

2) A second major topic on which there was less agreement was the Title. One reviewer stated that in their opinion, the title of the paper is too far reaching; it should be more narrow/descriptive to reflect what the paper is specifically about. The other reviewer felt that if this article is intended to impact future research broadly, having a short, intriguing title is helpful, both from the point of view of the author and the journal. We leave it to you, the author, to decide on the best title.

3) One reviewer stated their main concern was about the Bayesian analysis perspective, where the message became murky. They read this in the context of an essay intended to provide guidance for students and other researchers. Had this simply been an opinion piece, this would be less problematic, but the piece is fairly pedagogical. One important point that the author argues is that “inattention to principles underpinning Bayesian statistics reduce[s] experimental rigor.” This section on Bayesian inference would benefit from a more extensive exploration, including the description of Figure 5. The text is a bit cryptic, as the table in this figure is not explained effectively. The statement “the thought experiment presented in Figure 5 indicates that alpha seriously underestimates the proportion of false positive findings” does not adequately introduce these calculations. Moreover the + vs. - categories (e.g. “True +”) in the table are not explained. What do the blue values in this table signify?

4) Most importantly, what is the recommendation for how to proceed from this section on the Bayesian framework? The example in Fig. 5 is cautionary, but there is no guidance provided. How should a student or postdoc alter their statistical conclusions? This is particularly a dilemma when there is no good estimate available for the percent of hypotheses that are correct, as is frequently the case. (This may be less of an issue with high throughput screens or gene expression studies, although these estimates would vary with the assays for screens or the molecular process being analyzed.)

5) As far as data in the figures are concerned, Fig 2B is a replotting of published data from 2017, and Figs. 1, 2A and 3 are published data. These are not primary data. For Fig. 4, there are 43 respondents, but we are not told clearly how many received the problem. The same comment applies to the kidney cancer problem. It would be helpful to include data on demographics. More information is needed on the demographics of the survey respondents; a table would be helpful here. How many people received the survey and what was response rate? How were the responses to questionnaire items statistically or otherwise analyzed?

But given this is not truly a research paper, the requirements for data presentation can be relaxed.

6) Also related to the above. The results section is not traditional for an empirical paper. It appears to be a mix of qualitative and quantitative analyses of responses to three of the questionnaire items. Were these items selected for reporting from a larger set in the questionnaire for a particular reason? Assuming there were other items in the questionnaire, why are the responses to those items not reported? Some paragraphs in this section are not results, e.g., those that distinguish between exploratory and definitive experiments.

7) Page 8: “A set of anonymous survey questions was distributed to colleagues in several basic science departments at Emory University School of Medicine, and the results tabulated. It could aid understanding if the reader answers each question before reading on.” I don't understand what the second sentence means. Is it directed to the reader of this paper or to the survey respondents? What questions are referred to here?

8) p 14: “Respondents were asked to rank order the following nine fields by the likelihood that Chris is in that field (1=most likely).” What is other end of the scale? It is also not clear how this questionnaire item is related to “ignoring likelihood of a new result”. I am not sure how to interpret the graph in Fig 4. What does the outlier (#3 on the 1973 ranking) mean?

9) For the problem of predicting Chris's field of employment, were respondents shown employment rate data prior to their responding? In the case of Kahneman and Tversky, employment rate came from subjective impressions from a completely independent group of subjects.

10) In discussing the early work of Kahneman and Tversky, the author states “the nature of decision-making often involves over-reliance on instinct, to a degree not explicitly recognized...” It is important to appreciate the importance of “instinct” and unconscious creative processes in the development of hypotheses, i.e. explanatory models. Many important concepts that ultimately are rigorously validated have arisen through intuition or non-linear thinking. Thus the critical thinking skills required to rigorously test hypotheses are quite distinct from the cognitive processes involved in the initial generation of models. Moreover, it is our ability to see patterns in noisy preliminary data that enables us to formulate hypotheses.

11) The Abstract should emphasize that the Kahneman experiment repeated by the author(s) differs from the original study in having subjects who are researchers, rather than undergraduates across all disciplines. Therefore the similarity between the two sets of data is particularly striking. This difference should also be emphasized in the legend or axes for Figure 4; it's not the 4+ decades between studies that's so remarkable, but rather that the scientists in the recent study are presumably “critical thinkers.” Note, the subjects in the current study did preform twice as well on the “The Law of Small Numbers” problem (problem #1), which should be mentioned in the summary.

12) The list of opinions from postdoctoral fellows, students and research technicians concerning the obstacles to successful research listed as bullet points in the box at the beginning of Results is not well integrated into the rest of this essay. What is the point? Are the authors suggesting that these respondents entirely missed important factors that reduce the reliability of conclusions?

13) “Circumvention of cognitive biases is important in preclinical animal trials, cell culture experiments, any experiment in which outcomes are compared among two or more experimental groups.” There are actually few types of basic research studies where “circumvention of cognitive biases” is not essential. Once we appreciate that less preferred results may be excluded as outliers and essential controls may be omitted, the potential impact of bias seems ubiquitous across various types of experiments. Thus, this statement makes little sense.

14) Please elaborate on what a “single question psychology” project means, and how the questionnaire used in the current study was designed based on this approach. The author states in a couple of different places that the current study is a replication of Kahenman and Tversky's work. Does this mean the same questionnaire and methods of analysis were used, and only the respondents were different? Is this really a replication in the strict sense of the word? What specific cognitive constructs are questionnaire items designed to assess, and does the author have evidence for construct validity for each item? The questionnaire should be provided in its entirety so that the reader can fully evaluate what was asked of the respondents.

15) p 13: “Ignoring the likelihood of a new result”. Is this subheader at same level as The Law of Small Numbers and Intuitive Pattern Seeking? It is formatted differently (bolded, not underlined).

16) “an hypothesis” should be “a hypothesis.”

17) Although “scientific exploits” is catchy, it is probably not the best term in an article on improving scientific rigor.

18) Fig 1 needs a reference
